# An Influence of Modification with Phosphoryl Guanidine Combined with a 2′-O-Methyl or 2′-Fluoro Group on the Small-Interfering-RNA Effect

**DOI:** 10.3390/ijms22189784

**Published:** 2021-09-10

**Authors:** Anna S. Pavlova, Kristina I. Yakovleva, Anna V. Epanchitseva, Maxim S. Kupryushkin, Inna A. Pyshnaya, Dmitrii V. Pyshnyi, Elena I. Ryabchikova, Ilya S. Dovydenko

**Affiliations:** Institute of Chemical Biology and Fundamental Medicine (ICBFM), Siberian Branch of Russian Academy of Sciences (SB RAS), 630090 Novosibirsk, Russia; annivany@gmail.com (A.S.P.); yakovleva.kristinaa@yandex.ru (K.I.Y.); annaepanch@gmail.com (A.V.E.); kuprummax@gmail.com (M.S.K.); pyshnaya@niboch.nsc.ru (I.A.P.); pyshnyi@niboch.nsc.ru (D.V.P.)

**Keywords:** modified siRNA, phosphoryl guanidine group, RNA interference

## Abstract

Small interfering RNA (siRNA) is the most important tool for the manipulation of mRNA expression and needs protection from intracellular nucleases when delivered into the cell. In this work, we examined the effects of siRNA modification with the phosphoryl guanidine (PG) group, which, as shown earlier, makes oligodeoxynucleotides resistant to snake venom phosphodiesterase. We obtained a set of siRNAs containing combined modifications PG/2′-O-methyl (2′-OMe) or PG/2′-fluoro (2′-F); biophysical and biochemical properties were characterized for each duplex. We used the UV-melting approach to estimate the thermostability of the duplexes and RNAse A degradation assays to determine their stability. The ability to induce silencing was tested in cultured cells stably expressing green fluorescent protein. The introduction of the PG group as a rule decreased the thermodynamic stability of siRNA. At the same time, the siRNAs carrying PG groups showed increased resistance to RNase A. A gene silencing experiment indicated that the PG-modified siRNA retained its activity if the modifications were introduced into the passenger strand.

## 1. Introduction

RNA interference (RNAi) is a natural mechanism for regulating the expression of protein-coding genes in most of the eukaryotic cells. The regulation is mediated by the inhibition of mRNA translation by the enzymatic machinery of RNAi and small noncoding RNAs [1]. The involvement of small interfering RNAs (siRNAs) in the RNAi pathway leads to translation inhibition due to the degradation of a homologous mRNA target [2]. In mammalian cells, siRNAs are the product of cleavage of longer double-stranded-RNA precursors by an RNase III endonuclease called Dicer [3]. After the discovery of the ability of chemically synthesized siRNAs to induce RNAi in mammalian cells, these compounds have become a powerful tool for molecular biology, biotechnology, and functional genomics [4]. Good activity at nanomolar concentrations is the advantage of siRNAs that makes them the most promising type of therapeutic oligonucleotides among others aimed at inhibiting translation. Nonetheless, two decades after Fire and Mello discovered RNAi [5,6], only two siRNAs are approved for therapeutic use. The first RNAi-based therapeutic agent is ONPATTRO^®^ (patisiran, ALN-TTR02), which is siRNA-containing lipid nanoparticles for the treatment of polyneuropathies resulting from an inherited disorder, transthyretin-mediated amyloidosis [7]. The second therapeutic is GIVLAARI™ (givosiran, ALN-AS1), which is siRNA conjugated with N-acetylgalactosamine [8,9]. Widespread biomedical application of siRNAs is prevented by their low resistance to nucleases, their off-target effects, and the inability to independently penetrate into target cells. The idea to improve siRNA properties by chemical modifications has aroused much interest among researchers, and a number of different modifications have been proposed. The RNA structure determines the localization of chemical modifications that can be placed in an internucleotide phosphate, ribose moiety, or nucleoside [10]. As a rule, one type of modification is not sufficient to improve the properties of a particular siRNA, according to reports on patisiran and givosiran [9]. Thus, the development of new chemical modifications for nucleic acids and investigation of their properties when new modifications are combined with known ones remain relevant and important.

**Figure 1:**
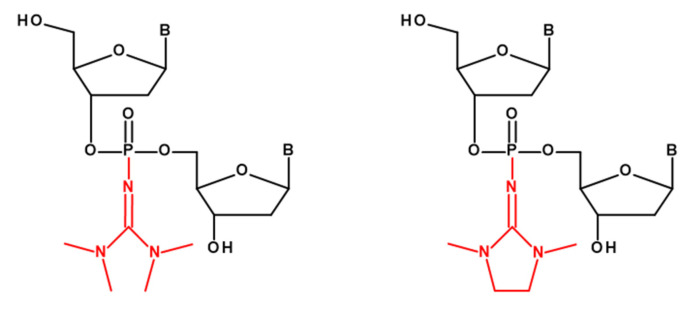
Examples of structures of mono-substituted phosphoryl guanidine (shown in red) dinucleotides.

Several years ago, at our institute, a new type of chemical modification of an internucleotide phosphate in oligonucleotides was developed, which was called a phosphoryl guanidine (PG) group (Figure 1) [11].

Oligonucleotides exhaustively modified with PG groups have complete resistance to nucleases; fully modified oligonucleotides have been shown to be resistant to snake venom phosphodiesterase [12]. It has been found that the presence of PG groups in the DNA chain of a chimeric DNA/RNA duplex does not change its conformation as compared with the native DNA/RNA analog; each PG group in the modified DNA/RNA duplex reduces the thermal stability of the duplex by an average of 1.2 °C, and this value does not depend on ionic strength of the solution [13].

In the present study, we report the properties of siRNA duplexes containing PG groups. These functionalized siRNAs were studied regarding thermal stability, RNAse A resistance, and the ability to induce RNAi in cultured mammalian cells in comparison with the corresponding unmodified (native) siRNA.

## 2. Results and Discussion

### 2.1. Design of PG-Modified siRNA

**Figure 2:**
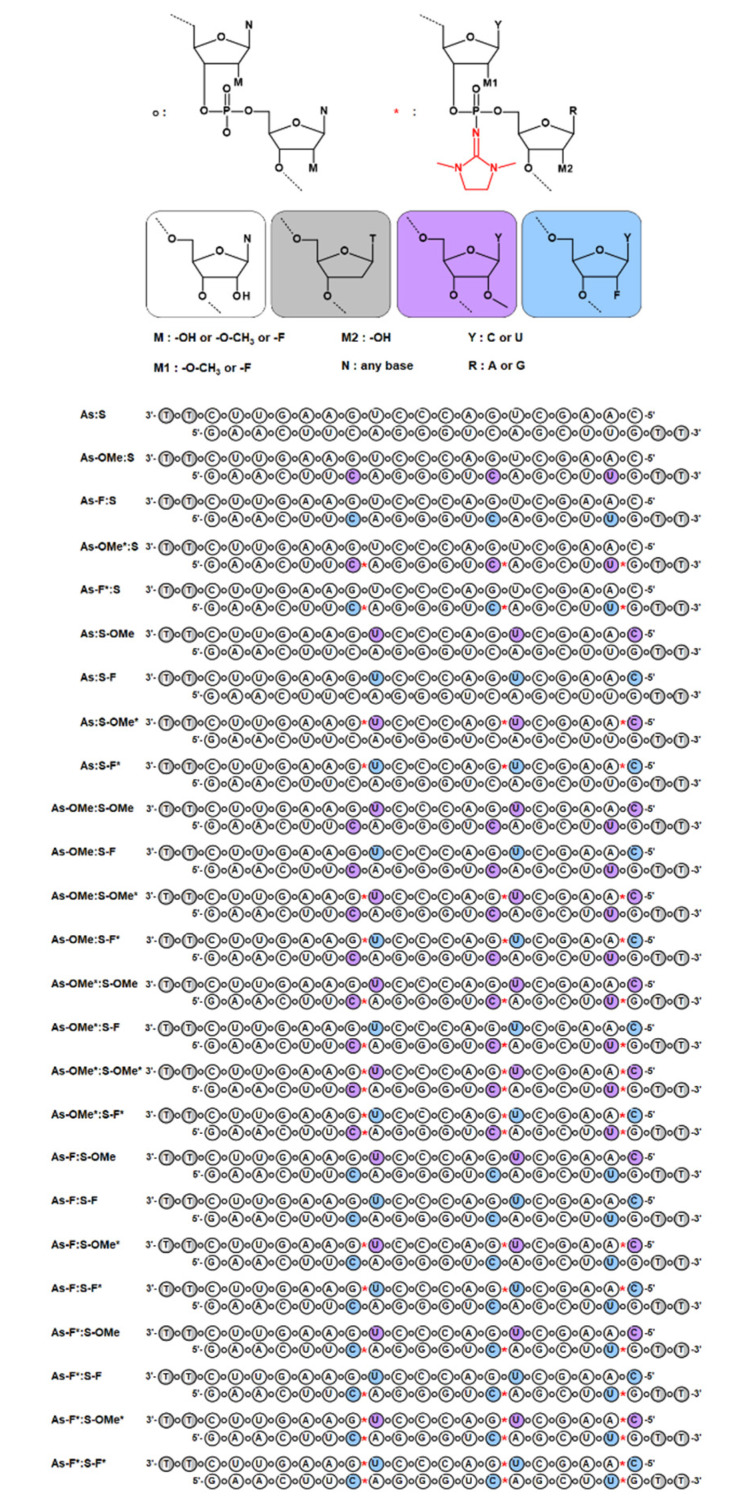
The library of the obtained duplexes: the unmodified siRNA and siRNAs bearing combined modifications PG/2′-OMe and PG/2′-F or containing only 2′-OMe and 2′-F substitutions. **As**: the antisense chain in an siRNA, **S**: the sense chain; * represents a PG-modified phosphate group.

At the first stage of the research, siRNA selection and design were carried out. The study is based on a single native siRNA sequence capable of silencing a stably expressed green fluorescent protein (*GFP*) gene [14]. Of note, it is impossible to obtain PG-modified oligoribonucleotides, because during the standard procedure of removal of a silyl protective group from the 2′-O position, the chain undergoes degradation at sites containing the PG group [15]. To avoid degradation of the oligoribonucleotide during postsynthetic treatments, it was decided to introduce PG groups into internucleotide phosphates where the ribose residue contained a modification at the 2′ position. Chemical modifications at the 2′ position of a ribose moiety with either oxymethyl (2′-OMe) or fluorine (2′-F) were chosen. Modifications 2′-Ome and 2′-F (separately) increase the thermal stability of a duplex [16,17]; therefore, we can compensate for the duplex destabilization caused by the PG group. Combinations of PG with 2′-OMe or 2′-F yielded oligoribonucleotide derivatives stable under deprotection conditions. It was decided to introduce the combined modifications PG/2′-OMe or PG/2′-F into the oligoribonucleotide chain only at certain sites. Even though complete modification of an oligoribonucleotide provides resistance to nucleases, complete modification with PG groups can inhibit RNAi. This is because PG is a rather bulky group and can introduce steric hindrances during the assembly of the RNA-induced silencing complex (RISC), thereby preventing RISC formation. Therefore, we chose to introduce modifications only into nuclease-sensitive siRNA sites (CA, UA, and UG) [18]. Each chain of siRNA contained three such sites; moreover, the nuclease-sensitive sites in the duplex were placed toward the 3′ end of the antisense chain. We assumed that in this way, we would be able to maintain the thermal asymmetry of the duplex so that the “correct” siRNA chain was loaded into the RISC [19,20]. To test our assumptions, we synthesized unmodified oligoribonucleotides as well as sense and antisense siRNA chains containing combined modification PG/2′-OMe or PG/2′-F or only 2′-OMe or 2′-F. By means of the synthesized oligoribonucleotides, an siRNA library consisting of 25 duplexes was prepared (all based on a single native sequence; Figure 2). For all the duplexes, comparative analyses of thermostability, resistance to RNAse A, and RNAi efficiency were carried out.

### 2.2. Duplex Thermostability

**Figure 3:**
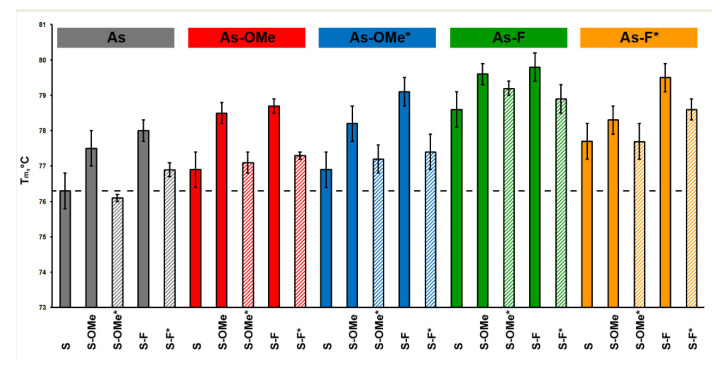
Melting temperatures (T_m_) of the siRNA duplexes. Boxes in the upper row describe modifications in the antisense chain (As) of an siRNA series. Colored vertical columns represent the data for each member of a series (each siRNA). For the convenience of representing different series of siRNAs, different colors were used. Striped columns denote PG-modified siRNAs. The asterisk (*) means the PG-modified chain.

The influence of PG substitutions on siRNA stability was analyzed by UV-melting experiments. Complementary duplexes of all pairs of sense and antisense oligoribonucleotides were formed under selected salt concentrations (ionic strength was close to physiological). The melting temperature (T_m_) of the duplexes varies from 76.1 to 79.8 °C (Figure 3).

The introduction of 2′-OMe or 2′-F substitutions led to an increase in T_m_ of the duplexes. The presence of the PG group in all cases destabilized the duplex, and the destabilizing effect of the PG group was not uniform and depended on a nucleotide sequence context. Let us consider the sense and antisense siRNA strands: the passenger strand contains two modified U nucleotides and one C, while the guide strand has the opposite set of modified nucleotides: two Cs and one U (Figure 2). Depending on which chain the combined modification PG/2′-OMe or PG/2′-F is located on, we observed a difference in the destabilization of the duplex from an analogous duplex that did not contain the PG group at the same position (Table 1).

Replacement of an internucleotide phosphate in the sense chain of an siRNA duplex by the PG group led to a greater destabilizing effect (Table 1, compare the following rows: the 1st with 11th, 2nd with 12th, 3rd with 13th, and so forth). Thus, A/U pairs, where U carries combined modification PG/2′-OMe or PG/2′-F, are more prone to this effect. It should be noted that the presence of combined modification PG/2′-OMe in the antisense chain either did not change the stability of the duplex or increased it relative to the analogous one containing only a methoxy group at 2′ positions of the antisense chain (Table 1, rows 11, 13, 15, 17, and 19). Nevertheless, this effect was absent when combined modifications PG/2′-OMe were located on the sense chain (Table 1, rows 1, 3, 5, 7, and 9). Perhaps the reason is that PG/2′-OMe, on the contrary, stabilizes a G/C pair. In any case, the presence of PG/2′-F always decreased the melting point of a duplex. Apparently, the difference between combined modifications PG/2′-OMe and PG/2′-F lies in the changes they cause to an RNA conformation (RNA helix); this difference requires additional structural research on these duplexes.

### 2.3. Stability of Modified siRNA in the Presence of RNase A

**Figure 4:**
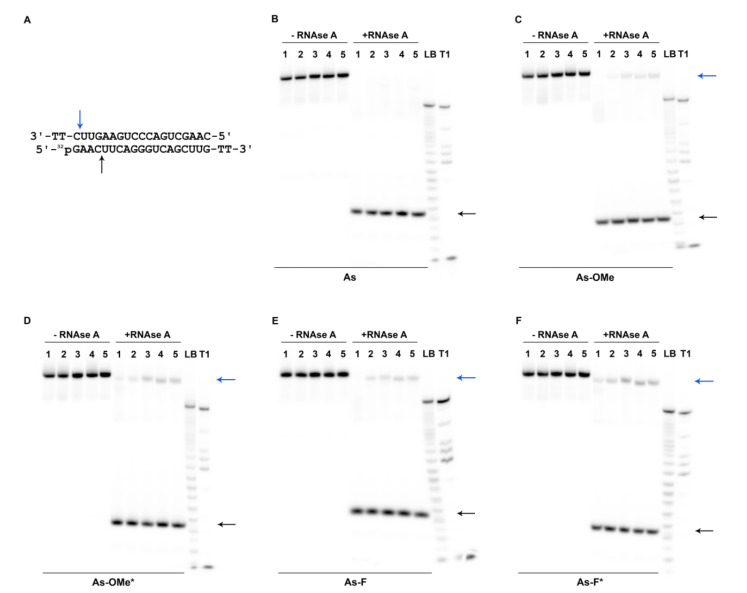
The products of degradation of the siRNA duplexes after 7.5 µg/mL RNAse A treatment, according to denaturing polyacrylamide gel electrophoresis (PAGE). (**A**). The black arrows indicate cleavage sites C_4_ identified in the ^32^P-labeled antisense strand; blue arrow: U_18_ in the unlabeled sense chains of the duplex. (**B**–**F**). Autoradiographs of 20% PAAGs with the corresponding ^32^P-labeled antisense chains; **+RNase A**: duplexes treated with RNase A; **-RNAse A**: control (undigested) duplexes. Each sample contained 1.5 pmol of ^32^P-labeled and 50 pmol of FITC-labeled antisense chains and 55 pmol of unlabeled sense chains. Antisense strands are designated at the bottom of each autoradiograph (B: As, C: As-OMe, D: As-OMe*, E: As-F, and F: As-F*)**. Lane 1** corresponds to the native sense (**S**) chain in all autoradiographs. Other lanes represent a modified sense chain: **Lane 2**: S-F, Lane 3: S-F*, Lane 4: **S-OMe**, and **Lane 5**: S-OMe*. After electrophoresis, the gels were dried, and Imaging Screen-K was exposed to the gels. Images were captured with PharosFX^TM^ Plus Molecular Imager^®^ (Bio-Rad, Hercules, CA, USA). Lanes **LB** and **T1** represent stochastic hydrolysis in 50 mM NaHCO_3_–Na_2_CO_3_ buffer (pH 9.5) and partial digestion of ^32^P-labeled As with RNase T1, respectively. The asterisk (*) means the PG-modified chain.

To evaluate the resistance of the obtained duplexes to a nuclease (RNase A), antisense-strand-labeled oligoribonucleotides were used. Oligoribonucleotides were labeled with ^32^P by means of a 5′-phosphate or fluorescent group (fluorescein isothiocyanate; FITC) at the 3′ end. To form a duplex, a mixture of fluorescently and radioactively labeled antisense oligoribonucleotides was employed. The siRNA duplexes were digested for 2 h with RNase A (7.5 µg/mL, 37.5 U) at 37 °C. After the reaction was stopped, the products of duplex degradation were analyzed by electrophoresis in a 20% denaturing polyacrylamide gel (PAAG). Since all the duplexes had high thermal stability, the long double-stranded fragments remaining after the digestion did not get denatured during the electrophoretic analysis. Accordingly, both double- and single-stranded fragments were present in the electropherograms (Figure 4).

**Figure 5:**
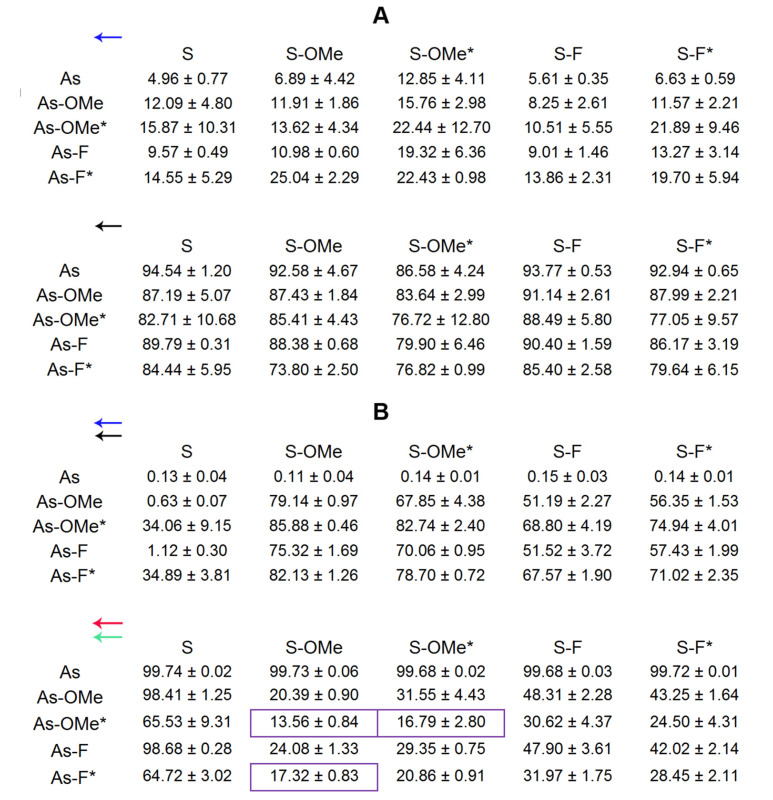
Cleavage levels (%) of siRNAs by RNAse A, as determined in autoradiographs ((**A**), two blocks) and fluorescent scanning gel images ((**B**), two blocks). The leftmost column in each block shows types of antisense chains; upper rows indicate types of sense chains. Each block contains data on the cleavage extent of a particular siRNA site. The blue arrow in the upper block of (**A**) means that this block shows the extent of cleavage of the U_18_ site in the sense chains of the duplex. The black arrow in the bottom block of (**A**) means that this block presents the extent of cleavage of the C_4_ site in an antisense strand. The double blue-and-black arrow in the upper block of (**B**) means that this block shows the extent of cleavage of the U_18_ site in sense chains and of the C_4_ site in antisense chains. The double red-and-green arrow in the bottom block of (**B**) means that this block shows the extent of cleavage of U_12_ and C_16_–U_17_ sites in antisense chains. The most stable siRNAs are boxed (violet frames). The extent of the degradation was calculated as the ratio of the intensity of a given band to the total intensity of all bands in a lane, multiplied by 100%. A lower value means lower degradation. Relative error in all the experiments did not exceed 20%. The asterisk (*) means the PG-modified chain. The data are presented as mean ± standard deviation from at least three independent experiments.

Figure 2 illustrates that the modifications in the antisense chain were placed closer to the 3′ end, while in the sense strand, they were closer to the 5′ end. Thus, the modifications in both chains overlap and are located closer to one side of the duplex. Due to this asymmetry, substantial degradation of the duplexes from the 5′ end of the antisense chain was observed (Figure 4B–F or Figure 5A; black arrow).

The sites of siRNA cleavage by RNAse A were determined via a comparison of band mobility of degradation products with bands resulting from stochastic hydrolysis (LB) and the pattern of antisense chain cleavage by RNAse T1 (T1). This assay revealed that a possible site of degradation in native and modified guide strands is located at the internucleotide phosphate group after nucleotide C_4_ (hereinafter, the subscript denotes a nucleotide position in an oligoribonucleotide sequence starting from position 1 at the 5′ end). It is located near the main RNAse A-preferred site: C_7_A_8_ (Figure 4A; black arrow) [21]. RNAse A hydrolyzed the siRNAs at the C_4_ position with high efficiency (Figure 5A, values under the black arrow). Given that the cleaved fragment is four nucleotides long, obviously, we see it as a single-stranded product in the autoradiographs (Figure 4B–F; black arrow). The products of cleavage at C_4_ containing ^32^P-labeled 5′-phosphate are characterized by identical mobility in the gel for all types of duplexes (Figure 4B–F, black arrow). Consequently, none of the degradation products contains a PG group because this group greatly changes the mobility of the fragment owing to its hydrophobicity and elimination of the charge.

We noticed the accumulation of an additional minor fragment with slightly higher mobility in the gel as compared to the full-length duplex after the introduction of modifications into the duplex-forming chains (Figure 4C–F, blue arrow). The reason for the greater mobility of this minor cleavage product compared to the full-length duplex could be cleavage at U_18_ at the 3′ end of the sense chain (Figure 4A; blue arrow). The absence of modifications at this position could lead to its quick complete cleavage. It should be noted that there was a correlation between the amount of the minor cleavage product and the bulkiness of modification at the C_7_A_8_ position of an antisense chain. The introduction of modification PG/2′-OMe or PG/2′-F caused greater accumulation of the minor band (Figure 5A, values under the blue arrow). Evidently, steric hindrances caused by PG groups negatively influence the cleavage kinetics of the neighboring C_4_ site.

**Figure 6:**
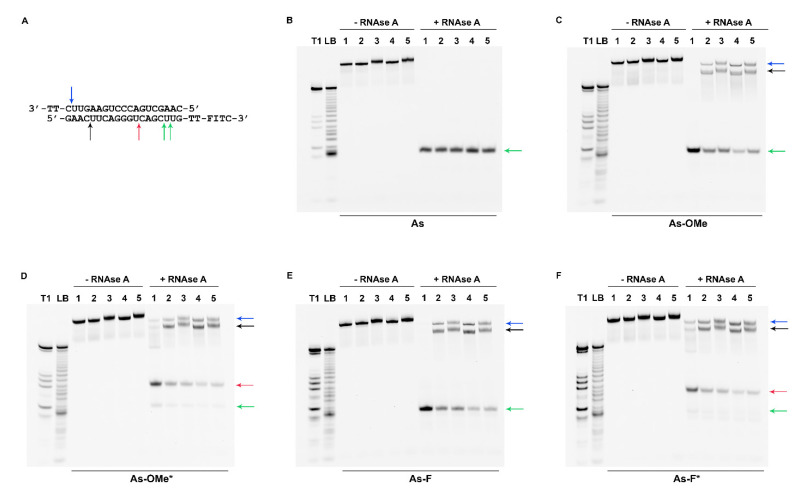
The products of siRNA duplex degradation after 7.5 µg/mL RNAse A treatment, according to denaturing PAGE. (**A**). The black arrow indicates cleavage sites C_4_ identified in an FITC-labeled antisense strand; red arrow: U_12_ in an FITC-labeled antisense strand; green arrow: the C_16_ or U_17_ site in an FITC-labeled antisense strand; blue arrow: U_18_ in the unlabeled sense strand of the duplex. (**B**–**F**): Gel images of 20% PAAGs with corresponding FITC-labeled antisense chains; **+RNase A**: duplexes treated with Rnase A; **-RNAse A**: control (undigested) duplexes. Each sample contained ^32^P-labeled and FITC-labeled antisense chains of the same structure (1.5 and 50 pmol, respectively) and 55 pmol of an unlabeled sense chain. Antisense strands are designated at the bottom of each image (B: As, C: As-Ome, D: As-Ome*, E: As-F, and F: As-F*). **Lane 1** contains a native sense (**S**) chain in all images. Other lanes contain a modified sense chain: **Lane 2**: S-F, Lane 3: S-F*, Lane 4: **S-Ome**, and **Lane 5**: S-Ome*. The products of degradation of FITC-labeled siRNAs duplexes were visualized in the gel via scanning and recording of an image using a VersaDoc^TM^ MP 4000 Molecular Imager^®^ System (Bio-Rad) after laser excitation at 488 nm. Lanes **LB** and **T1** present the stochastic hydrolysis in 50 mM NaHCO_3_–Na_2_CO_3_ buffer (pH 9.5) and partial digestion of FITC-labeled As with Rnase T1, respectively. The asterisk (*) means the PG-modified chain.

Additionally, the formation of a large number of truncated fragments was registered after the cleavage of duplexes bearing a fluorescent label at the 3′ end. The mobility and amount of cleavage products varied among the duplexes (Figure 6B–F; red and green arrows).

**Figure 7:**
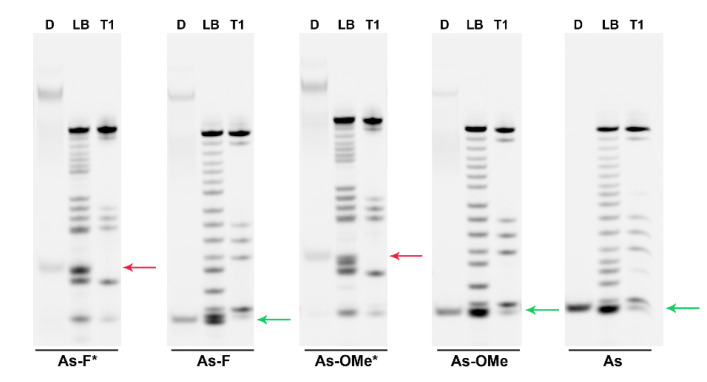
Cleavage site determination in FITC-labeled modified siRNAs by denaturing PAGE. Electropherograms for products of degradation of five siRNAs after 7.5 µg/mL RNAse A treatment. Each image has a **D** lane representing an RNAse A-treated duplex that contains S-OMe* and various derivatives of the antisense chain (As-F*, As-F, As-OMe*, **As-OMe**, or **As**). The products of degradation of FITC-labeled siRNA duplexes were visualized in the gel via scanning and recording of an image by means of the VersaDoc^TM^ MP 4000 Molecular Imager^®^ System (Bio-Rad) after laser excitation at 488 nm. Lanes **LB** and **T1** are the stochastic hydrolysis in 50 mM NaHCO_3_–Na_2_CO_3_ buffer (pH 9.5) and partial digestion of the FITC-labeled As with RNase T1, respectively. The asterisk (*) means the PG-modified chain.

The mobility rates were identical among the most truncated fragments in the series of duplexes with the native antisense chain or with As-OMe or As-F (Figure 6B,C,E; green arrow). According to the T1 and LB ladders, the cleavage in these duplexes could occur between nucleotides C_16_ and U_18_ (Figure 6A–C,E; green arrows). Then, we identified the cleavage site more precisely, C_16_–U_17_, owing to higher resolution in case of As-F (Figure 7, As-F, green arrow). The introduction of modifications into the sense chain reduced the efficiency of the duplex cleavage (Figure 6B,C,E; lines 2–5, Figure 5B, values under the green arrow). In this case, degradation products with lower mobility appeared in the electropherograms: two bands below the control duplex (Figure 6B,C,E; blue and black arrows). Apparently, these two degradation products derive from duplexes in which the phosphodiester bonds were cleaved after the C_4_ position in the guide strand and after U_18_ in the passenger strand, as revealed by the cleavage assay of the duplexes containing an antisense chain bearing ^32^P-labeled 5′-phosphate.

Modification of an antisense chain with PG resulted in an additional product of cleavage (Figure 6D,F; red arrow). A comparison of degradation products with the T1 and LB ladders obtained by means of PG-containing oligoribonucleotides confirmed the change in the cleavage site after the U_12_ nucleotide as a consequence of the modification (Figure 7, red arrow).

The cleavage at positions C_16_-U_17_ of As-Ome or As-F and at U_12_ of As-Ome* or As-F* (Figure 6, green and red arrows) occurred at a site adjacent to modified nucleotides. Considering this, the cleavage extent at these sites should be the lowest in the duplexes whose modifications protect against RNAse A most strongly. These most stable duplexes (As-OMe*/S-OMe, As-F*/S-OMe, and As-OMe*/S-OMe*) are highlighted in Figure 5B by violet frames.

Our results suggested that the introduction of PG groups substantially protects a duplex at the 3′ end of the antisense chain and can cause RNAse A to change its position of interaction with the siRNA. Apparently, this is because the PG group either creates steric hindrances for RNase A or provokes a change in the duplex conformation. In this regard, some of the internucleotide phosphates adjacent to the modified site become inaccessible to the active center of the nuclease.

Thus, we demonstrated that it is possible to obtain stable siRNA derivatives through modification with PG. A question remains: How will PG influence RISC function in the cell?

### 2.4. The Activity of Modifed siRNA In Vitro

The ability of the synthesized duplexes to induce RNAi was tested on cultured SC-1 cells stably expressing GFP. The cells were transfected with an siRNA lipoplex with the help of Lipofectamine 3000 in a serum-free medium; the final siRNA concentration was 50 nM. After 4 h of incubation of the cells with the lipoplex, fetal bovine serum (FBS) was added to 10% (*v*/*v*), and the cells were incubated for another 12–16 h. Then, the medium was refreshed (without the lipoplex), and the transfected cells were grown for another 3 days to degrade the previously accumulated GFP.

**Figure 8:**
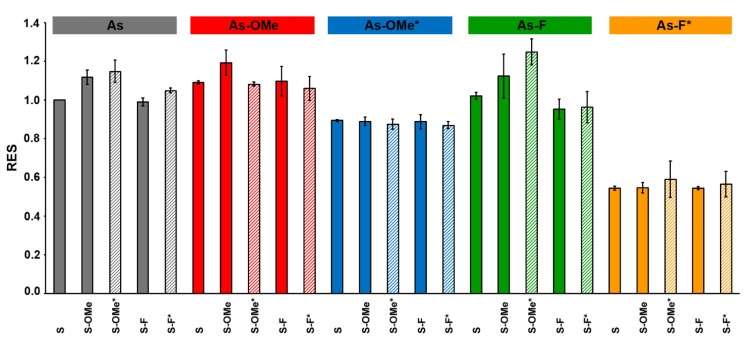
Relative efficiency of silencing (**RES**) determined for each siRNA. Boxes in the upper row describe modifications in the antisense chain (As) of an siRNA series. Colored vertical columns represent the data for each member of a series (each siRNA). For the convenience of representing different series of siRNAs, different colors were used. Striped columns denote PG-modified siRNAs. The asterisk (*) means the PG-modified chain.

Next, the cells were analyzed by flow cytometry: the fluorescent-signal intensity and the number of cells with the level of fluorescence of control cells (incubated with Lipofectamine 3000 only) were determined. The data obtained for each modified siRNA were normalized to the result obtained with the cells transfected with the native siRNA (As:S) from the same experimental series (Figure 8). Detailed explanation of the calculations is presented in Section 3.7.

For each duplex, three transfections were performed. The data (Figure 8) revealed that the silencing of GFP expression was the least effective for all duplexes containing the As-F* antisense chain, whereas duplexes containing only modification 2′-F in the antisense chain induced the silencing similarly to native siRNA. Duplexes containing the PG group were more resistant to the nuclease than the native analogs were, and the change in the siRNA activity may be explained by a number of factors, namely, a decrease in the efficiency of duplex formation between the target and the seed region of the antisense chain, impaired thermal asymmetry of an siRNA duplex, or obstacles to RISC formation [22].

Another series of siRNAs, containing combined modification 2′-OMe/PG in the antisense strand (As-OMe*), also had low efficiency of silencing but better efficiency than that of duplexes carrying As-F*. The melting temperature of the duplexes containing a PG group in the antisense chain is almost equal (or lower for As-F*) to that of similar duplexes without the PG group (Table 1). Therefore, disruption of the asymmetry of a duplex’s thermal stability or the formation of a complex target/seed region presumably is not the reason for the decrease in silencing efficiency. We assumed that the bulkiness of the PG group negatively affects RISC formation. On the other hand, the combination of 2′-OMe and PG is bulkier than 2′-F/PG, but the lowest silencing efficiency was registered for the combination with 2′-F. Obviously, the differences in efficiency between the duplexes containing 2′-OMe/PG and 2′-F/PG in the antisense chain are related to other factors. Probably, the proximity of fluorine to a PG-modified internucleotide phosphate leads to polarization of the latter [23], thereby completely disrupting the RISC. Nonetheless, the presence of the PG group in the sense chain did not reduce the efficiency of silencing. The duplexes containing a PG group only in the sense chain in combination with 2′-OMe also exerted a stronger silencing effect than did analogs carrying the 2′-F/PG combination (Figure 8). The cell culture experiments definitely showed that a PG group should be introduced only into the sense chain of siRNA to maintain RNAi.

## 3. Materials and Methods

### 3.1. Reagents

RNA phosphoramidites and solid supports for oligoribonucleotide synthesis were acquired from Glen Research (Sterling, VA, USA); FITC isomer I and other chemicals were supplied by Sigma-Aldrich (St. Louis, MO, USA). DNase- and protease-free RNAse A (10 mg/mL) was purchased from Thermo Fisher Scientific (Baltics UAB, Vilnius, Lithuania), and RNAse T1 of *Aspergillus oryzae* (ammonium sulfate suspension) 362000 U/mg protein, 0.53 mg protein/mL, was purchased from Sigma-Aldrich. The total RNA from *Escherichia coli* (100 μg/mL) was kindly provided by Dr. Nina A. Moor (ICBFM SB RAS). Lipofectamine 3000 was bought from Thermo Fisher Scientific. All other chemicals used in this work were of molecular biology grade or higher. Water was of 18 MΩ grade (purified in a Simplicity 185 water purification system; Millipore, Burlington, MA, USA).

### 3.2. Oligonucleotide Synthesis

Oligoribonucleotides were synthesized on an automatic DNA/RNA ASM-800 synthesizer (Biosset, Novosibirsk, Russia) at 0.4 mmol scale using 2′-O-*tret*-butyldimethylsilyl–protected, 2′-OMe, 2′-F, and 2′-deoxy phosphoramidites and solid-phase phosphoramidite synthesis protocols [24] optimized for the instrument. The synthesis of 3′-amino-modified derivatives of oligoribonucleotides was carried out on 3′-PT-Amino-Modifier C6 CPG (Glen Research). The cleavage of oligoribonucleotides from the support and removal of the nucleobase exocyclic amine were conducted in a 30% ammonia hydroxide/40% methylamine mixture (1:1) for 15 min at 65 °C; for oligoribonucleotides containing 2′-F substitutions, this procedure was performed at 25 °C for 2 h. Elimination of 2′-O-silyl protective groups was carried out as described previously [25]. The sequence of the siRNA targeting *GFP* mRNA was borrowed from ref. [14].

### 3.3. UV-Melting Experiments

To prepare duplexes of the siRNA, an equimolar mixture containing 2.5 µM of each RNA strand in a buffer consisting of 127 mM NaCl and 10 mM sodium cacodylate C_2_H_12_AsNaO_5_ (pH 7.2) was incubated for 3 min at 95 °C and then was cooled slowly to 25 °C. Thermal denaturation assays of the siRNA duplexes were conducted in quartz cells (0.2 cm path length) on a Cary 300 Bio UV-VIS spectrophotometer (Varian, Palo Alto, CA, USA) equipped with a Peltier thermostabilized multicell holder (6 × 6). Melting curves were recorded at wavelengths 260, 270, and 330 nm in the 5−90 °C range at a temperature change rate of 0.5 °C/min. Absorbance at 330 nm was subtracted from the values at other wavelengths at each temperature. T_m_ was calculated as a maximum of the first derivative of optical absorbance in terms of temperature. Each result was an average from two experiments on denaturation and renaturation at two wavelengths (260 and 270 nm).

### 3.4. 5′-End-^32^P-Labeling of Antisense siRNA Chains

Antisense siRNA chains (100 nmol) were 5′-^32^P-labeled for 1−2 h at 37 °C in a buffer (10 μL) consisting of 50 mM Tris-HCl pH 7.6, 10 mM MgCl_2_, 5 mM dithiothreitol, 50 μM (0.1 mCi) γ[^32^P]ATP, and 40 U of T4 polynucleotide kinase followed by precipitation and dissolving in 1× denaturing dye-containing buffer for electrophoresis (8 M urea, bromophenol blue, xylene cyanol). ^32^P-Labeled antisense siRNA chains were isolated by electrophoresis in a denaturing 15% PAAG (acrylamide (AA)/bis-acrylamide (BA) at 29:1) and TBE (89 mM Tris-borate pH 8.3, 2 mM EDTA) as running buffer. The RNAs were cut out of the gel and eluted three times with 300 μL of elution buffer (0.3 M LiClO_4_) followed by desalting on Bio-Spin^®^ 6 Columns (Bio-Rad); then, they were precipitated with 2% LiClO_4_ in acetone and stored until use at −20 °C.

### 3.5. 3’-End-Labeling of Antisense siRNA Chains with FITC

The labeling of 3′-amino-modified oligoribonucleotides with FITC was carried out as described previously [26]. Briefly, an oligonucleotide containing a 3′-terminal amino linker (5 OD units at A_260_) dissolved in 50 μL of 0.1 M HEPES-NaOH pH 8.5 was mixed with 50 μL of FITC dissolved in dry DMSO at 10 mg/mL. The reaction mixture was incubated overnight at room temperature with constant stirring. For subsequent purification, the conjugates were precipitated with ethanol as Na salts. FITC-labeled antisense siRNA chains were isolated by electrophoresis in a denaturing 15% PAAG (AA:BA at 29:1) and TBE as running buffer. The RNAs were cut out of the gel and eluted three times with 300 μL of elution buffer (0.3 M sodium acetate, pH 5.2), precipitated with ethanol, and stored at −20 °C until experiments.

### 3.6. RNAse A Degradation Assays

For the assembly of siRNA, a mixture of ^32^P-labeled and FITC-labeled antisense chains of the same structure (1.5 and 50 pmol, respectively), as well as 55 pmol of the unlabeled sense chain in a buffer composed of 50 mM Tris-acetate pH 7.5, 100 mM NaCl, and 0.2 mM EDTA was incubated for 3 min at 95 °C and then cooled slowly to 25 °C. The hydrolysis reaction was initiated by the addition of RNAse A to a final concentration of 7.5 µg/mL. The reaction mixtures were incubated for 2 h at 37 °C; the reaction was stopped by the addition of 0.01% (*w*/*v*) SDS, 1.5 M β-mercaptoethanol, and 1× denaturing dye-containing buffer for electrophoresis (8 M urea, XC) prior to loading onto a denaturing 20% PAAG (AA:BA at 29:1). After electrophoretic separation, the products of degradation of the FITC-labeled siRNA duplexes were visualized in the gel by scanning and recording an image by means of the VersaDoc^TM^ MP 4000 Molecular Imager^®^ System (Bio-Rad) after laser excitation at 488 nm. After that, the gels were dried, and Imaging Screen-K was exposed to the gels. Images containing the products of degradation of ^32^P-labeled siRNA duplexes were captured with a PharosFX^TM^ Plus Molecular Imager^®^ System (Bio-Rad). The signal intensity was quantified in the Quantity One software (Bio-Rad). The degradation extent was calculated as the ratio of the intensity of a given band to the total intensity of all bands in the lane, multiplied by 100%. The relative error in all experiments did not exceed 20%. Each type of siRNA duplex was tested in three or more independent experiments. The degradation extent is reported as mean ± standard deviation from at least three independent experiments.

Stochastic cleavage (LB) of siRNA chains was performed in 50 mM NaHCO_3_–Na_2_CO_3_ buffer (pH 9.5) for 10 min at 90 °C. After the addition of 1× denaturing dye-containing buffer (8 M urea, XC), the reaction mixture was analyzed by electrophoresis in a 20% PAAG (AA:BA at 29:1).

Cleavage of siRNA antisense chains with 5 AU RNase T1 (T1) was performed in a buffer composed of 6 M urea, 25 mM sodium citrate Na_3_C_6_H_5_O_7_ pH 4.8, 1 mM EDTA, and total RNA from *E. coli* (100 μg/mL). The sample mixtures were equilibrated for 28 min at 55 °C; next, the reaction was stopped by the addition of an equal volume of 2× TBE and 1× denaturing dye-containing buffer (8 M urea, XC).

The resulting sets of markers (LB and T1) were utilized to identify the cleavage products in the electrophoretic analysis.

### 3.7. Cell Culture and Transfection

Mouse embryonic SC-1 fibroblasts with stable integration of the *GFP* gene into the genome were kindly provided by Dr. V. S. Prassolov (Engelhardt Institute of Molecular Biology, RAS, Moscow). The cells were maintained at 37 °C in Dulbecco’s modified Eagle’s medium (DMEM) (Life Technologies, Paisley, UK) supplemented with 10% of heat-inactivated FBS (Life Technologies) and 1% of PeniStrep (Life Technologies, Grand Island, NY, USA) in a humidified atmosphere containing 5% of CO_2_.

For transfection experiments, 7 × 10^4^ cells were seeded in each well of 24-well tissue culture plates (TPP, Trasadingen, Switzerland). After 24 h of cultivation, the cells were washed with PBS, and an siRNA in complex with Lipofectamine 3000 (final concentration of siRNA: 50 nM) in DMEM (without FBS and PeniStrep) was added. The cells were cultivated for 4 h (at 37 °C and 5% CO_2_); then, 55 μL of FBS was added, and the cells were cultured in the presence of the transfectant for additional 12–16 h. After that, the medium was replaced with fresh DMEM supplemented with FBS.

Silencing efficiency was evaluated by flow cytometry on a NovoCyte instrument (ACEA Biosciences, San Diego, CA, USA). More than 10,000 cells from each sample were analyzed in the NovoExpress software (ACEA Biosciences). Each series of transfection experiments was repeated three times, and the absolute values varied slightly with identical differences between experiments. Simple averaging of the values was not correct, and we decided to employ the relative efficiency of each modified siRNA in comparison with the native siRNA.

To determine the relative efficiency of silencing (RES), firstly, a population of stand-alone control cells (treated with Lipofectamine 3000 only) was gated. The obtained gate masks were applied to data from each siRNA-treated group of cells. In this way, we determined a proportion of cells without silencing within each sample treated with an siRNA (*X_i_*, where *i* is the type of siRNA). Next, we calculated RES as *X_As:S_*/*X_i_*.

RES revealed changes in the efficiency with respect to the native siRNA: RES > 1 means a greater silencing effect relative to the native siRNA, and RES < 1 denotes a reduction or loss of the effect.

### 3.8. Statistical Analysis

Each experiment was performed independently three times for all assays. The data are presented as mean ± standard deviation from at least three independent experiments.

## 4. Conclusions

Our findings clarify the feasibility of using of PG groups for siRNA modification. The instability of the PG-modified internucleotide phosphate in the presence of 2′-OH nearby means that the PG group should be used only in combination with 2′-O-protected or 2′-substituted nucleotides. This limitation requires more scrupulous selection of the set of additional modifications that will be combined with the PG group because its presence influences the thermodynamic stability of the siRNA (Table 1).

The introduction of a combined modification improves the resistance of the siRNA to nuclease. This type of modification increases the resistance not only of the modified site but also of neighboring native internucleotide moieties. Most likely, this phenomenon is due to the nature of the PG group, which is a sufficiently bulky substituent. This group introduces serious steric hindrances so that the access of nucleases to neighboring sites is limited. This bulkiness also makes modifying the guide strand pointless, because the introduction of PG abrogates the silencing effect. Nevertheless, this feature may serve as a means of eliminating an off-target effect of the passenger strand unintentionally entering a RISC. Our results of cell culture experiments suggest that the modification with the PG group makes sense only for the sense chain of siRNA.

## Figures and Tables

**Table 1:** Comparison of melting points of duplexes containing a PG group (the name contains an asterisk) with the analogous duplexes without a PG group at the same positions. ΔT_m_ is the difference between the average melting points of the examined and comparison duplexes. The asterisk (*) means the PG-modified chain.

Row	Examined Duplex	Comparison Duplex	ΔT_m_, °C
1	As:S-OMe*	As:S-OMe	−1.4
2	As:S-F*	As:S-F	−1.1
3	As-OMe:S-OMe*	As-OMe:S-OMe	−1.4
4	As-OMe:S-F*	As-OMe:S-F	−1.4
5	As-OMe*:S-OMe*	As-OMe*:S-OMe	−1
6	As-OMe*:S-F*	As-OMe*:S-F	−1.7
7	As-F:S-OMe*	As-F:S-OMe	−0.4
8	As-F:S-F*	As-F:S-F	−0.9
9	As-F*:S-OMe*	As-F*:S-OMe	−0.6
10	As-F*:S-F*	As-F*:S-F	−0.9
11	As-OMe*:S	As-OMe:S	0
12	As-F*:S	As-F:S	−0.9
13	As-OMe*:S-OMe	As-OMe:S-OMe	−0.3
14	As-F*:S-OMe	As-F:S-OMe	−1.3
15	As-OMe*:S-OMe*	As-OMe:S-OMe*	0.1
16	As-F*:S-OMe*	As-F:S-OMe*	−1.5
17	As-OMe*:S-F	As-OMe:S-F	0.4
18	As-F*:S-F	As-F:S-F	−0.3
19	As-OMe*:S-F*	As-OMe:S-F*	0.1
20	As-F*:S-F*	As-F:S-F*	−0.3

## Data Availability

Data is available on request from the corresponding authors.
